# Supplementary material for: Effect of interfacial defects on the electronic properties of MoS2 based lateral T–H heterophase junctions
Source: RSC Adv. 2021 Nov 25;11(60):37995–8002. doi: 10.1039/d1ra06010d (PMC9044014; doi:10.1039/d1ra06010d)
Supplement: RA-011-D1RA06010D-s001 [file RA-011-D1RA06010D-s001.pdf]

Electronic Supplementary Material (ESI) for RSC Advances.
This journal is © The Royal Society of Chemistry 2021

Effect of Interfacial Defects on the Electronic Properties of MoS_2 Based Lateral T-H Heterophase Junctions

Mohammad Bahmani[1,†], Mahdi Ghorbani-Asl[2,‡], and Thomas Frauenheim[⋆,1,3,4]

[1] *Bremen Center for Computational Materials Science (BCCMS), Department of Physics, Bremen University, 28359 Bremen, Germany*

[2] *Institute of Ion Beam Physics and Materials Research, Helmholtz-Zentrum Dresden-Rossendorf, 01328 Dresden, Germany*

[3] *Beijing Computational Science Research Center (CSRC), 100193 Beijing, China*

[4] *Shenzhen JL Computational Science and Applied Research Institute, 518110 Shenzhen, China*

The coexistence of semiconducting (2H) and metallic (1T) phases of MoS_2 monolayers have further pushed their strong potential for applications in the next generation of electronic devices based on the two-dimensional lateral heterojunctions. Structural defects have considerable effects on the properties of these 2D devices. In particular, the interfaces of two phases are often imperfect and may contain numerous vacancies created by phase engineering techniques, e.g. under the electron beam. Here, the transport behaviors of the heterojunctions in the existence of point defects are explored by means of first-principles calculations and non-equilibrium Green's function approach. While vacancies in semiconducting MoS_2 act as scattering centers, their presence at the interface improves the flow of the charge carriers. In the case of V_{Mo}, the current has been increased by two orders of magnitude in comparison to the perfect device. The enhancement of transmission was explained by changes in the electronic densities at the T-H interface, which open new transport channels for electron conduction.

† mbahmani@uni-bremen.de, ‡ mahdi.ghorbani@hzdr.de, ⋆ frauenheim@uni-bremen.de

Structure of the devices containing point defects

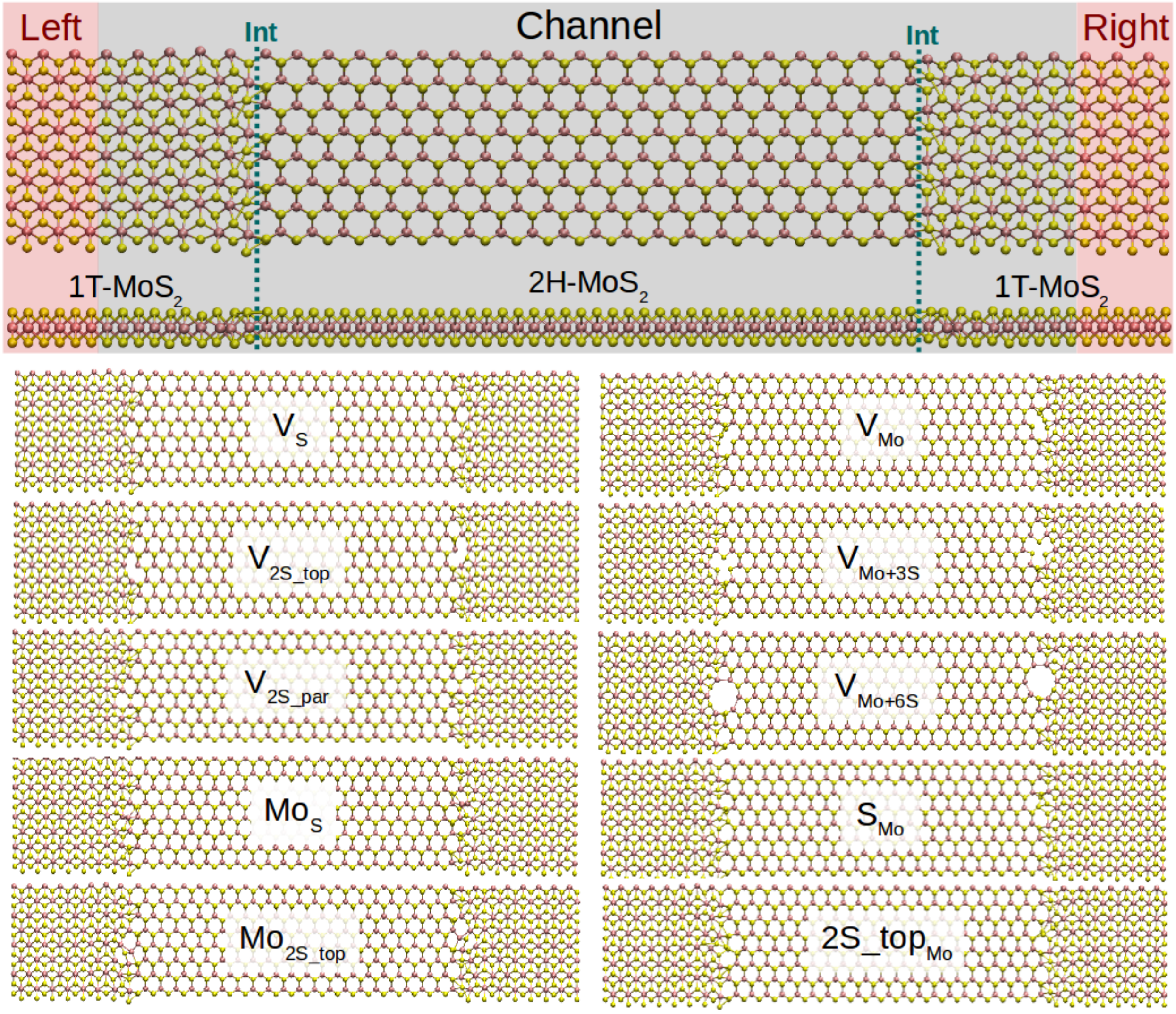

Figure S1: **Top)** Schematic of the device based on metallic (1T) and Semiconductor (2H) phases of MoS_2 monolayers is shown. Electrodes (only 1T-MoS_2) and channel region (a combination of 1T- and 2H-MoS_2) are highlighted with shaded red and black, respectively. The interfaces are indicated with green dashed-lines. All the structures are considered periodic along the axis transverse to the transport direction. **Down)** Optimized defective structures with point defects at both interfaces of the devices.

Band structure of 1T/2H phases of MoS_2 monolayers

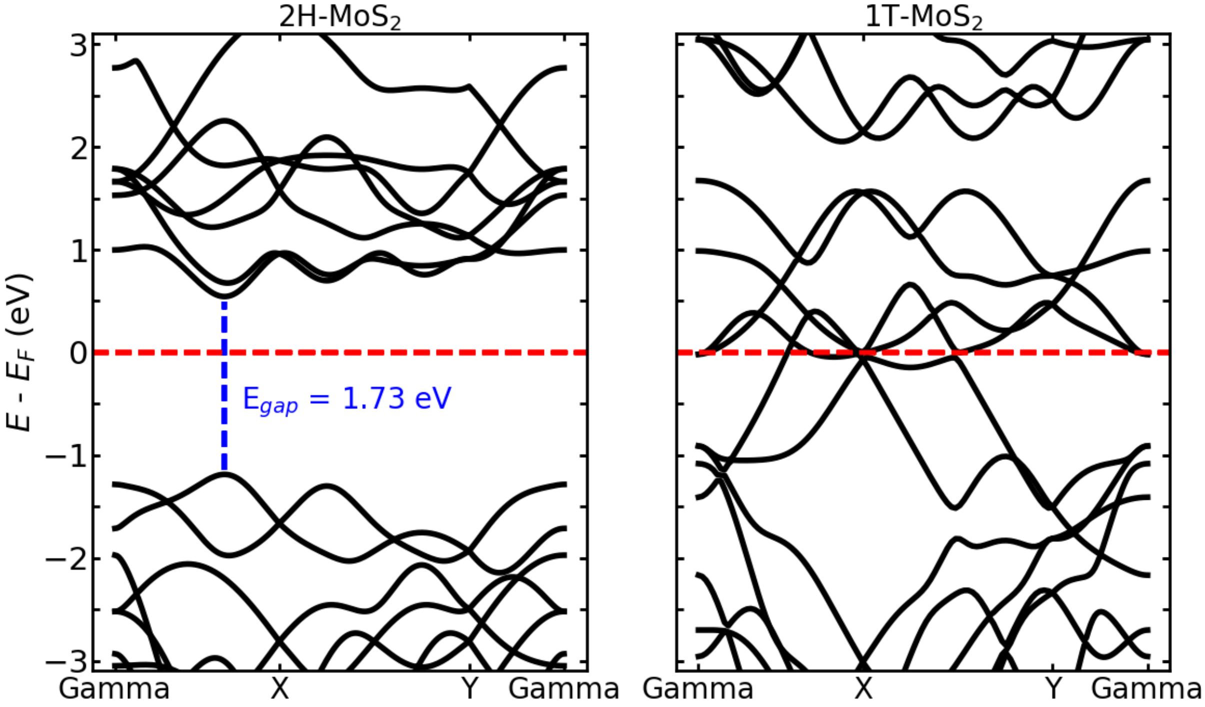

Figure S2: Band structures are plotted for **left)** Semiconductor (2H) and **right)** metallic (1T) phases of MoS_2 monolayers. Energies are shifted according to the Fermi energy. There is a bandgap of 1.73 eV for the case of the semiconducting monolayers while 1T-MoS_2 has a clear metallic character.

Displacement map of surrounding atoms

Shown in Fig. S3, the amount of displacement due to the absence of different combination of atoms are calculated to further study the effect of induced local strain on the device properties. Here, the displacements in all three axis are summed up to obtain the total local strain. It can be seen that the largest change in the atomic positions are for the case of V_{Mo+3S} at the interfaces while removing two Sulfurs, from top and bottom layers, induce the smallest displacements into the devices.

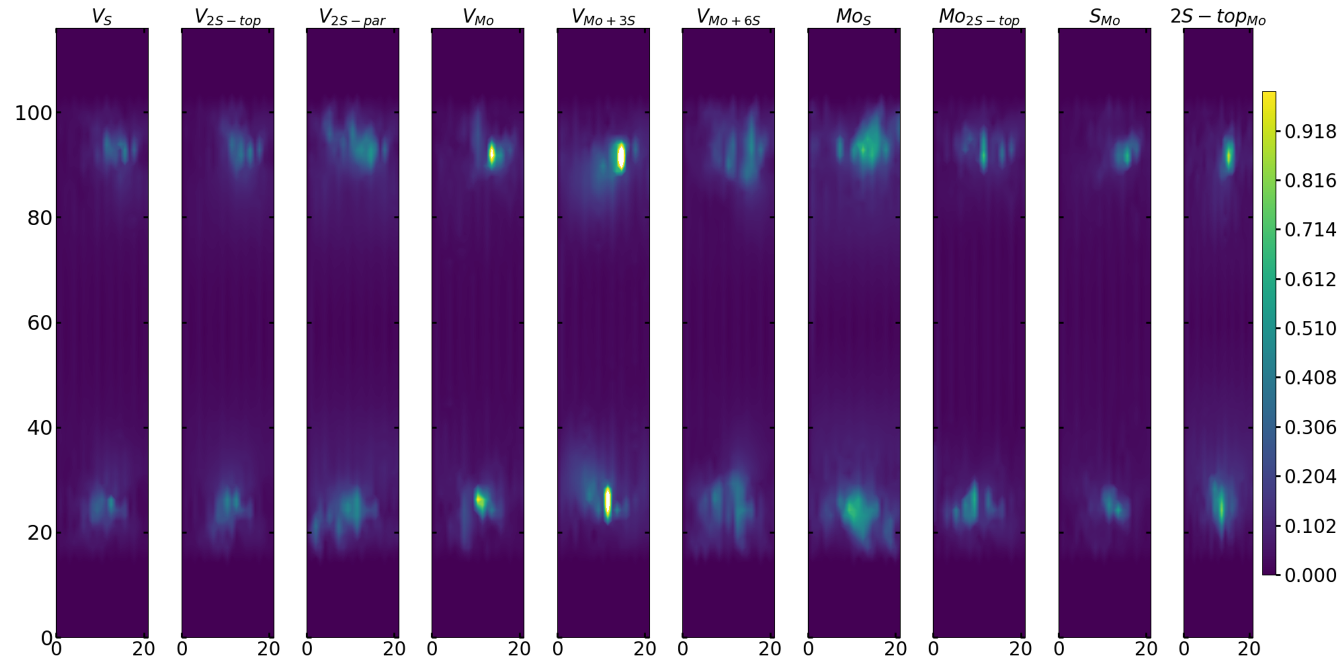

Figure S3: Displacement map of the defective structures. Sum of displacements in the three axis are calculated.

Vector Current - V_{Mo}

Shown in Fig. S4, vector currents are plotted for perfect systems and devices with V_{Mo} at both interfaces. Vector current displays the direction and the amount of current (size of the arrows) projected on each atom and at specific energy channel (E_{ch}), that comes from left (blue) or right (red) electrode. It can be clearly seen that the presence of the vacancy at the interfaces opens up further energy channels for the charge carriers to move.

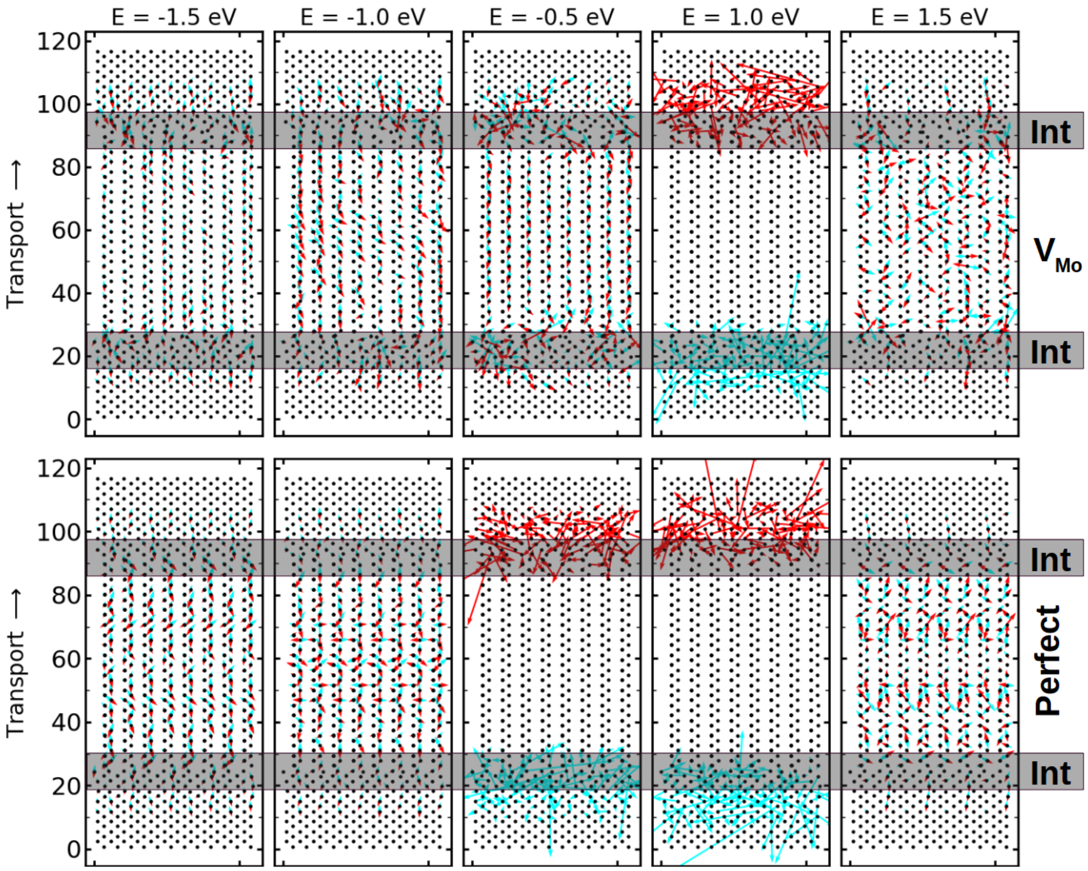

Figure S4: (Color online) Vector currents are shown for devices with perfect interfaces and where molybdenum vacancy, V_{Mo}, are present at both interfaces. They are plotted at zero bias and specific energy channels, namely E= -1.5, -1.0, -0.5, 1.0, $1.5 eV$. Blue and red arrows display the current at each atom that comes from left and right electrode, respectively. The length of the arrow indicates the current magnitude. It can be clearly seen that the presence of the vacancy at the interfaces opens up further energy channels for the charge carriers to move.

Local Density of States (LDOS) projected on atoms and orbitals

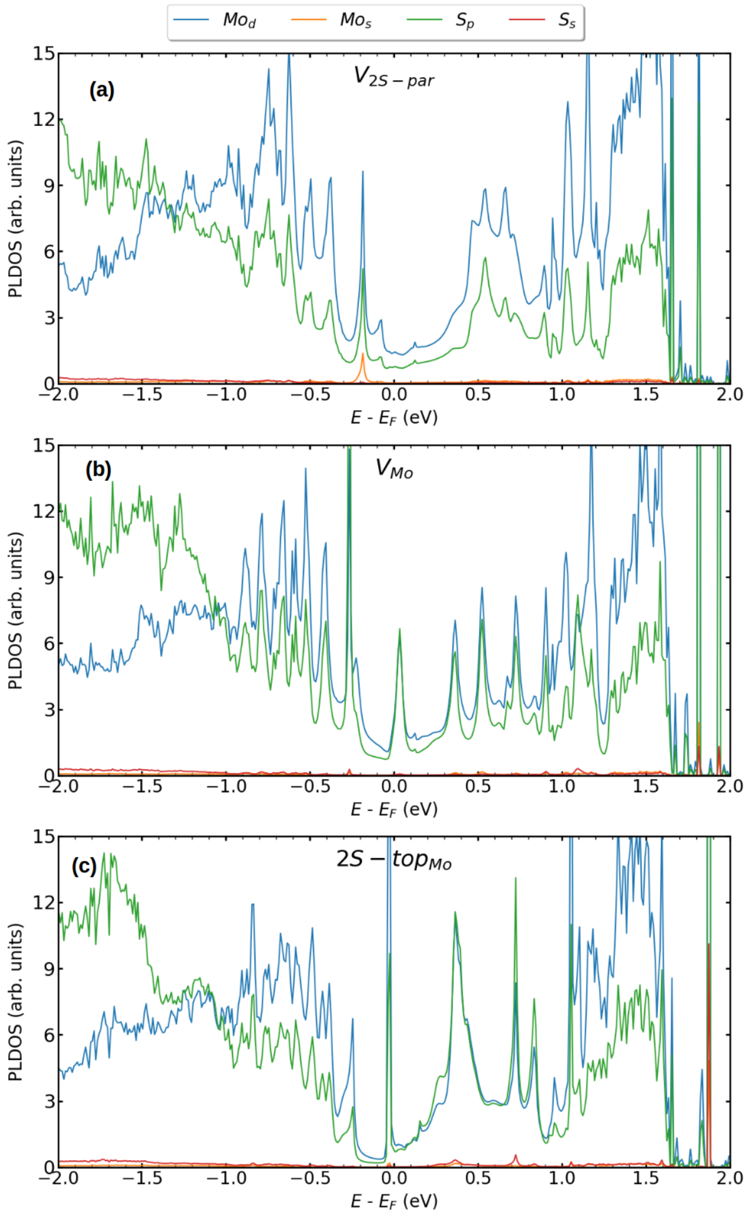

Figure S5: (Color online) Density of states of the Molybdenum and Sulfur atoms at the left interface projected (LDOS) onto the orbitals contributing to the total DOS for devices containing **a)** V_{2S-par}, **b)** V_{Mo}, **c)** $2S-top_{Mo}$, at both interfaces. It clearly shows that the most contributions come from the Mo_d and S_p orbitals.

Calculated BandGap vs. other methods

Method	This paper	PBE-GGA[a]	HSE[b]	GW[b]
BandGap (eV)	1.76	1.715	2.21	2.78

Table S1: Band gaps of 2H-MoS_2 monolayer calculated at different levels of theory. a) Rafael Roldn, et. al Annalen der Physik 526(9-10), 347357 (2014), b. H.-P. Komsa and A. V. Krasheninnikov, Physical Review B,2015, 91, c. C. Ataca and S. Ciraci, The Journal of Physical Chemistry C, 2011, 115, 1330313311

Projected Local Density of States (PLDOS)

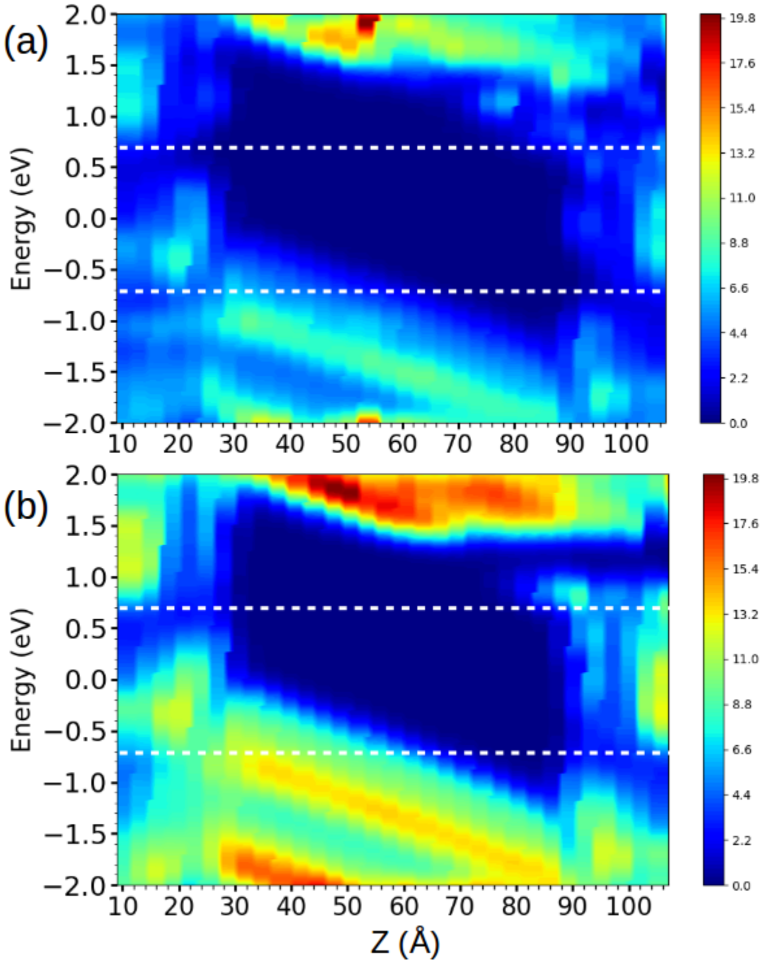

Figure S6: (Color online) Projected local density of states (PLDOS) of devices based on T-H heterophase junction of MoS_2 monolayer under the biases with a) perfect interface and b) containing molybdenum vacancy. The white dotted lines indicate the bias window.
